# On Clinical Agreement on the Visibility and Extent of Anatomical Layers in Digital Gonio Photographs

**DOI:** 10.1167/tvst.10.11.1

**Published:** 2021-09-01

**Authors:** Andrea Peroni, Anna Paviotti, Mauro Campigotto, Luis Abegão Pinto, Carlo Alberto Cutolo, Yue Shi, Caroline Cobb, Jacintha Gong, Sirjhun Patel, Stewart Gillan, Andrew Tatham, Emanuele Trucco

**Affiliations:** 1 VAMPIRE Project, Computing, School of Science and Engineering, University of Dundee, Dundee, UK; 2 NIDEK Technologies Srl, Albignasego, Italy; 3 Department of Ophthalmology, Hospital Santa Maria, Lisbon, Portugal; 4 Clinica Oculistica, Di.N.O.G.M.I., University of Genoa, Genoa, Italy; 5 Doheny Image Reading Center, Doheny Eye Institute, Los Angeles, CA, USA; 6 Department of Ophthalmology, Ninewells Hospital, NHS Tayside, Dundee, UK; 7 Princess Alexandra Eye Pavilion, NHS Lothian, Edinburgh, UK

**Keywords:** inter-annotator variability, automated gonioscopy, AI software validation

## Abstract

**Purpose:**

To quantitatively evaluate the inter-annotator variability of clinicians tracing the contours of anatomical layers of the iridocorneal angle on digital gonio photographs, thus providing a baseline for the validation of automated analysis algorithms.

**Methods:**

Using a software annotation tool on a common set of 20 images, five experienced ophthalmologists highlighted the contours of five anatomical layers of interest: iris root (IR), ciliary body band (CBB), scleral spur (SS), trabecular meshwork (TM), and cornea (C). Inter-annotator variability was assessed by (1) comparing the number of times ophthalmologists delineated each layer in the dataset; (2) quantifying how the consensus area for each layer (i.e., the intersection area of observers’ delineations) varied with the consensus threshold; and (3) calculating agreement among annotators using average per-layer precision, sensitivity, and Dice score.

**Results:**

The SS showed the largest difference in annotation frequency (31%) and the minimum overall agreement in terms of consensus size (∼28% of the labeled pixels). The average annotator's per-layer statistics showed consistent patterns, with lower agreement on the CBB and SS (average Dice score ranges of 0.61–0.7 and 0.73–0.78, respectively) and better agreement on the IR, TM, and C (average Dice score ranges of 0.97–0.98, 0.84–0.9, and 0.93–0.96, respectively).

**Conclusions:**

There was considerable inter-annotator variation in identifying contours of some anatomical layers in digital gonio photographs. Our pilot indicates that agreement was best on IR, TM, and C but poorer for CBB and SS.

**Translational Relevance:**

This study provides a comprehensive description of inter-annotator agreement on digital gonio photographs segmentation as a baseline for validating deep learning models for automated gonioscopy.

## Introduction

Gonioscopy^1^ enables visual inspection of the iridocorneal angle (ICA), the anatomical region in the anterior chamber of the eye where aqueous humor drainage occurs through the trabecular meshwork, regulating intraocular eye pressure. If aqueous outflow is diminished, intraocular eye pressure may rise, thus increasing the risk of developing glaucoma,^2^ an irreversible optic neuropathy and one of the main causes of blindness worldwide.^3^ Common types of glaucoma include open-angle glaucoma, where aqueous outflow is hampered by increased resistance through the trabecular meshwork, and angle-closure glaucoma, where outflow resistance occurs due to appositional or synechial contact between the iris and the trabecular meshwork. Although, worldwide, open-angle glaucoma is more common, angle-closure glaucoma is responsible for a disproportionate number of patients with severe vision loss.^4^ Gonioscopy is thus fundamental to assessing the status of the ICA and identifying eyes with, or at risk of, angle closure.^5^^,^^6^

The current clinical standard technique for gonioscopy is a slit-lamp–assisted examination using a contact lens, which requires significant time, patient cooperation, and operator expertise. The result is that gonioscopy is often performed less frequently than recommended^7^ and is seldom practiced by optometrists in primary-care settings, with implications for identifying those at risk of angle closure. Gonioscopy is subject to considerable interobserver variability, and it is also difficult to obtain images for the patient record, thus reducing the ability to verify diagnosis and detect changes over time, such as increasing peripheral anterior synechiae.

Anterior-segment ocular coherence tomography allows the acquisition of cross-sectional images of the ICA region; it requires less experience and causes less discomfort to patients. However, it does not provide a direct, complete visualization of the ICA interface and does not allow the observer to see peripheral anterior synechiae or evaluate trabecular meshwork pigmentation, which is a significant ICA feature.

The recent availability of new semi-automatic imaging devices^8^^,^^9^ for gonioscopy has the potential to address current limitations and offers an unprecedented opportunity for the development of automated image analysis software, such as machine and deep learning algorithms, to support assisted diagnoses or to present augmented data to clinicians^10^ (Cappellari L, et al. *IOVS*. 2020;61:ARVO E-Abstract 1620). Crucially, reliable ground truth (i.e., image annotations) must be generated in order to tune and validate automatic systems.

In general, annotations, such as anatomical layer contours, are approximations of properties of the real structures that are impossible to obtain directly. It is well known^11^^–^^13^ that inter-annotator variability affects annotations consistency and, in turn, protocols for validation and overall performance assessment. Modeling annotation variability is an established and fundamental requisite for software validation.

We present, to the best of our knowledge, the first inter-annotator variability study on manual delineations of ICA layers in digital gonio photographs.

Promising results have been obtained recently with deep learning for automatic angle closure classification in digital gonio photographs based on the visibility of the pigmented trabecular meshwork.^14^ We argue that the assessment of other clinically relevant features could benefit from a local, rather than global, characterization of the ICA anatomy (i.e., a pixel-wise classification or segmentation). A precise delineation of ICA layers could be advantageous, for example, for measuring synechial closure extension and its changes over time or segmenting the trabecular meshwork to allow automatic pigmentation grading (e.g., prior to laser trabeculoplasty). Moreover, auto-alignment and auto-tracking systems based on layers segmentation could improve examinations in remote and virtual clinics, which are currently gaining importance due to the COVID-19 pandemic.

## Methods

### Data Acquisition

Digital gonio photographs (1280 × 960 pixels RGB) of the ICA interface were acquired using a NIDEK GS-1 semi-automatic gonioscope (NIDEK Co., Ltd., Gamagori, Japan) at three European clinical sites located in Genova, Italy; Lisbon, Portugal; and Dundee, United Kingdom. The NIDEK GS-1 takes multiple color images of the ICA to cover the entire 360° of the interface at different focal planes with limited depth of field. Each image covers a 22.5° wide sector.

Data were acquired with patients’ agreement and in accordance with the General Data Protection Regulation. Acquisition conditions may vary according to physicians’ discretion but without upsetting image quality for the purposes of our analysis.

After anonymizing the data at the source, 20 sector images were selected from 18 eyes of 17 patients and were used to study inter-annotator variability. For each sector considered, the image with the focus on the edge of the ICA (i.e., on the ciliary body band or the scleral spur if the angle sector was open or on the iris–cornea interface if it was closed) was selected to provide the sharpest (highest contrast) visualization of the layer interfaces. The limited depth of field implies that the inner portion of the iris and the outer portion of the cornea may appear blurred. A vignette is also visible in most images, whereby the image periphery appears darker than its center.

Image selection was not based on acquisition conditions or the patient's diagnosis (e.g., ocular hypertension, glaucoma) but only on local layers morphology, as this study aimed to assess inter-annotator variability on descriptive image features and not to relate these features to diagnosis. A clinical stratification of patients is, thus, not relevant and is not provided.

Rather, the images selected are representative for a range of variations of the ICA features observed in clinical practice, such as iris color and trabecular meshwork pigmentation, and include relevant local variations of layers interfaces, such as appositional angle closure and anterior synechiae. More in detail, the images include six light and 14 dark irises (where blue or green eyes were considered light and brown eyes were considered dark); five highly and 11 slightly pigmented trabecular meshworks in non-closed angle sectors (where slightly pigmented corresponds to Scheie's pigmentation grades none, 1, and 2, and highly pigmented corresponds to Scheie's grades 3 and 4); four angle-closure images defined as appositional iridocorneal contact in at least 50% of the sector (Scheie's grade 4); and four images showing anterior synechiae.

### Data Annotation Protocol

A comprehensive annotation protocol was designed in collaboration with the ophthalmologists participating in this study. The interdisciplinary team ensured that the information provided by the annotations was both clinically meaningful and useful to potentially train and validate automatic deep learning systems ranging from layer detection to semantic segmentation purposes.

Here, “to annotate” means to trace the contours of the layers visible in the image and assign them the correct label. The annotation tool we selected was the VGG Image Annotator 2.0.8.^15^ Image regions were highlighted using polygonal shapes, and labels were selected from a list of available entries.

**Figure 1.**
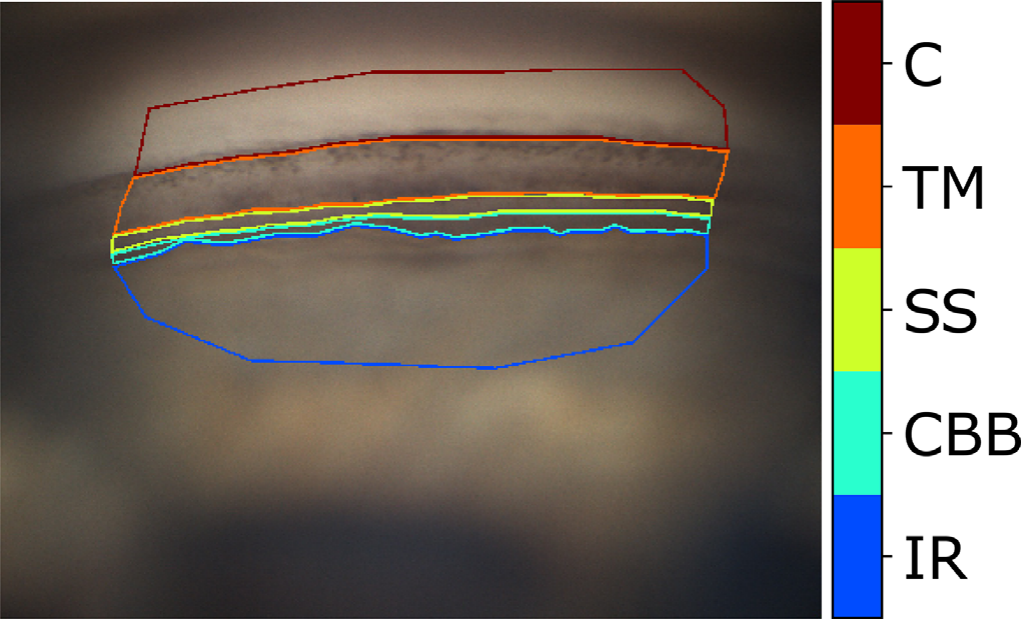
An example of annotated sector image. IR, iris root; CBB, ciliary body band; SS, scleral spur; TM, trabecular meshwork; C, cornea. (Gonio photograph courtesy of C.E. Traverso, MD, Clinica Oculistica Di.N.O.G.M.I., Ospedale San Martino, Genoa, Italy.)

Figure 1 shows an example of annotated sector image.

The annotation protocol stipulates to annotate only the sharpest (in-focus) and well-lit image areas; to not trace the contours of target layers that were not clearly identifiable with respect to neighboring one; and to trace layer–layer interfaces as precisely as possible. Although this protocol means the annotations are suitable for validating automatic segmentation systems, it does not necessarily reflect what is commonly done by ophthalmologists in clinical practice; for example, tracing contours of ICA layers is not usually required.

Five ophthalmologists from four clinical institutions in Genoa, Italy; Lisbon, Portugal; Dundee, United Kingdom; and Los Angeles, California, USA, provided annotations. They were trained individually in the use of the annotation tool and protocol, in person whenever possible and through web meetings, digital documentation, and online support otherwise. Annotators could access the entire exam acquisition for every sector image selected to take advantage of all of the information available. When they performed the annotations, SP and JG were, respectively, year 4 and 7 specialty trainees with experience in gonioscopy; YS was a clinical study investigator with 5 years of experience in an image reading center; CAC was a glaucoma specialist with 5 years of clinical experience in glaucoma management; and LAP was head of the Glaucoma Section, with more than 10 years of clinical experience in tertiary referral centers.

### Annotations Characteristics

Annotations consisted of a set of non-overlapping polygonal contours enclosing the best-lit and sharpest area of each ICA layer considered in this study: iris root (IR), ciliary body band (CBB), scleral spur (SS), trabecular meshwork (TM), and cornea (C).

Image characteristics and protocol guidelines had important consequences on the annotations. For example, even if layers are present over the whole image, vignetting and blur might result in annotators ignoring the image periphery. This means that a degree of subjectivity was always present, such as in locating the transition between in-focus and blurred regions of the iris or the transition between well-lit and dark regions within the trabecular meshwork. Moreover, annotators might choose not to annotate part of an image if they did not feel sufficiently confident (e.g., if they judged the region too poor from a qualitative point of view). This did not necessarily indicate disagreement with the other annotators, and these premises resulted in part of each image being left un-annotated (labeled NA). Each clinician annotated at least one anatomical layer in every image.

In our analysis, inter-annotator variability only accounted for annotated image regions and was never affected by un-annotated areas. This ensured that variability measures reflected only differences in clinical considerations made with confidence.

**Figure 2.**
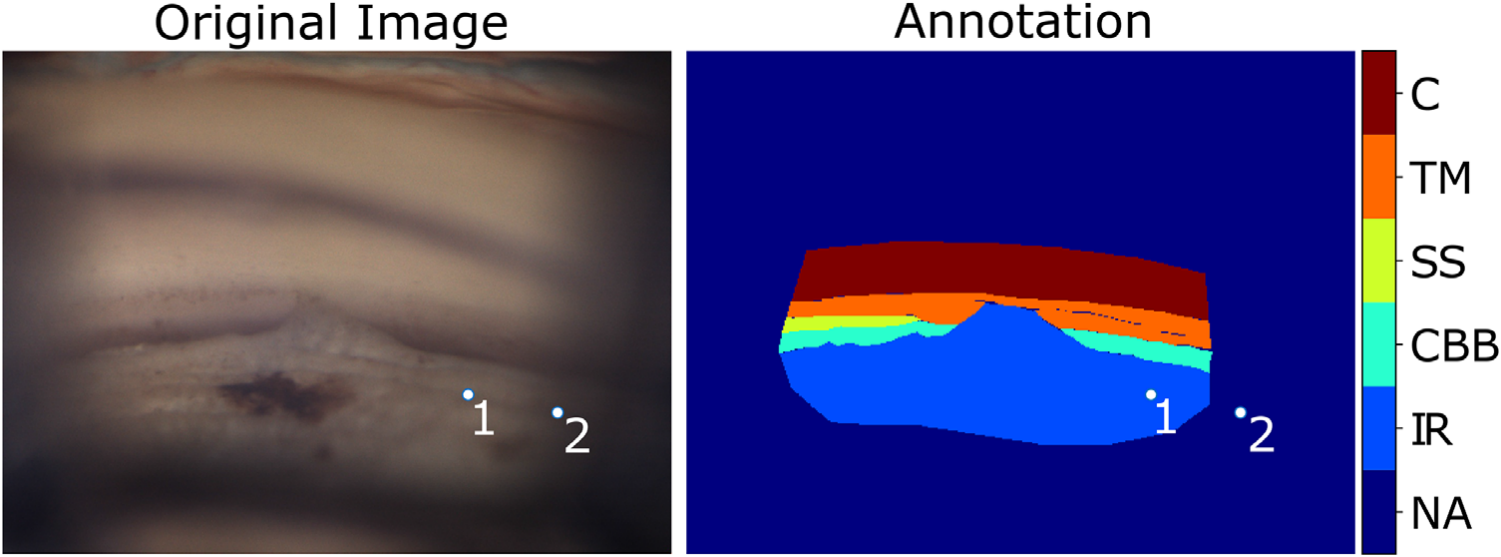
Original gonio photograph (*left*) and an annotation (*right*). Points 1 and 2 highlight two pixels in the iris root. Point 2 has been excluded from the annotation, given the subjective estimation of the transition between the well-lit and dark image regions. (Gonio photograph courtesy of C.E. Traverso, MD, Clinica Oculistica Di.N.O.G.M.I., Ospedale San Martino, Genoa, Italy.)

An example of digital gonio photograph and an annotation thereof is shown in Figure 2. All of the image pixels within a delineation were labeled as pertaining to that layer. The two pixels highlighted in the figure belong to the same ICA layer (iris root), but the point labeled “2” in the image was not included in the annotated region, given the (subjectively) estimated border between well-lit and dark regions.

This is a departure from many inter-annotator variability studies for segmentation systems in medical image analysis,^16^^,^^17^ which typically expect the entire contour of targets to be identifiable. These characteristics mean that standard analysis methods, such as consensus, and comparison metrics, such as the Dice score, required adapting in order to be used consistently.

### Inter-Annotator Variability Analysis

Inter-annotator variability was analyzed in three experiments, reported below.

#### Layer-Wise Annotation Frequency

Layer-wise annotation frequency refers to the number of times each clinician delineated the contours of each structure, as a measure of their confidence at recognizing and locating ICA layers in digital gonio photographs. Annotators were instructed to trace contours only when they judged them to be clearly visible. Occasionally, some layers were not visible at all; for example, the scleral spur was not visible in the case of angle closure. For this experiment, we were only interested in the existence of a layer annotation, not in its geometry; hence, two annotators could be equally confident in delineating a specific layer even if the two actual contours differed. This methodology was designed to provide insight into the variability in experts’ confidence in identifying ICA layers from local image features of digital gonio photographs.

#### Layer Consensus As a Function of the Number of Agreeing Annotators

This analysis examined consensus by the number of pixels agreed to be part of a given layer by a minimum number of annotators. Its size was plotted as a function of the minimum number of agreeing annotators (consensus threshold). The purpose was to obtain an indication of which layers were annotated with high and low consistency among the annotators in terms of location and size.

In the literature,^11^ the consensus of multiple annotations of the same image is usually computed as the subset of pixels labeled in the same way by at least *n* (consensus threshold) annotators, with all of the other pixels considered as background.

We adapted this concept for our study to deal correctly with the un-annotated areas (i.e., not background), defining a three-category label for each layer pixel, as follows:

1. Consensus region (label 1)—The set of pixels annotated as the given layer by at least *n* observers.
2. Disagreement region (label –1)—The set of pixels annotated as the given layer by *k* annotators, with *1 ≤ k < n*, and differently (i.e., belonging to another layer) by at least one.
3. Ignored region (label 0)—The set of pixels annotated as the given layer by *k* annotators, with *k < n*, and left un-annotated by the others; this region is ignored when computing consensus size variations, as its variability does not necessarily reflect changes of the actual agreement level.

It follows that NA image regions do not affect consensus computation according to our experimental design.

**Figure 3.**
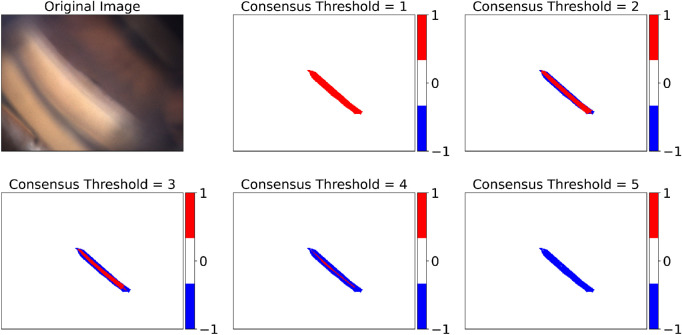
Original RGB image (*top left*) and the five scleral spur consensus maps as the consensus threshold varies. Label 1 is the consensus region, –1 is the disagreement region, and 0 is the ignored region. (Gonio photograph courtesy of C.E. Traverso, MD, Clinica Oculistica Di.N.O.G.M.I., Ospedale San Martino, Genoa, Italy.)

An example of how the consensus region varied with the consensus threshold is shown in Figure 3. The extent of disagreement increased with the consensus threshold value, as expected. In the ideal case of perfect agreement among all the annotators, the agreement region size would not change varying the threshold.

After generating five consensus maps for each target layer, one per threshold value (when the threshold equaled 1 it led to the union of annotated pixels, and when it equaled 5 it led to their intersection), we studied how the consensus size decreased as the threshold increased.

#### Average Per-Layer Agreement Analysis

This analysis compared agreement between pairs of annotators, with one annotator chosen as reference for each pair.

Each comparison yielded a 5 × 5 confusion matrix, given that there were five target classes. Un-annotated regions were excluded from the computation so that intersections between areas that were annotated by one annotator but not by the other one did not affect the results.

Layer-wise precision, sensitivity, and Dice scores of each annotator were calculated as follows:

1. Precision—*TP/*(*TP + FP*), where *TP* is the true positives and *FP* is the false positives.
2. Sensitivity—*TP/*(*TP + FN*), where *FN* is the false negatives.
3. Dice score—2 × (*precision* × *sensitivity*)*/*(*precision + sensitivity*)

Average values and standard deviations of each annotator were computed as an overall measure of inter-annotator agreement.

## Results

### Layer-Wise Annotation Frequency

**Figure 4.**
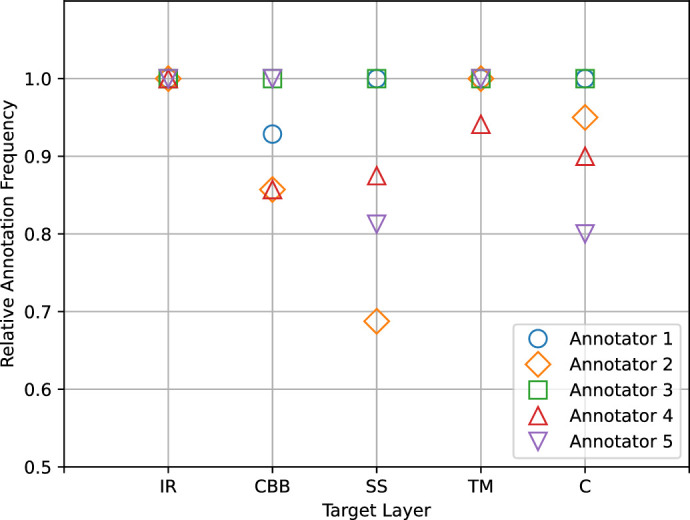
Plot of per-layer annotation frequencies for each annotator.

Figure 4 shows the per-layer annotation frequency of each annotator, a measure of how confidently they identified and traced contours. Note that the fixed sequence of the layers provides expectations about what layers are present in a given location, but segmentation (contours) depends on local image features.

The iris root was the only region segmented by all participants the same number of times (i.e., most consistently). This can be explained by considering that the boundary between the iris and the next visible layer is usually sharp and thus well identifiable, but this may not be true for other layers. The relative segmentation frequency measured for the remaining layers varied, up to a maximum percent difference of 31% for the scleral spur (annotator 2 vs. annotators 1 and 3).

Only one participant (annotator 3) provided the observed maximum number of annotations for all of the layers.

### Layer Consensus As a Function of the Number of Agreeing Annotators

**Figure 5.**
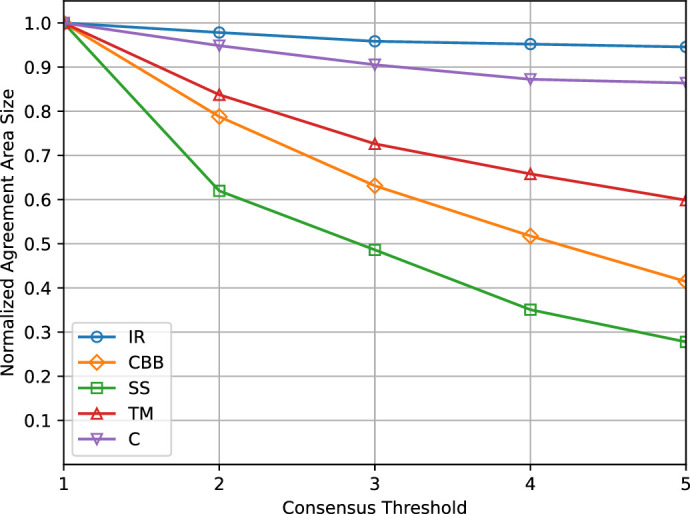
Plot of the ratio between consensus pixels and annotated pixels against the consensus threshold (minimum number of annotators agreeing).

Figure 5 shows how the area of the consensus region for each layer, normalized to (0, 1), decreased as the consensus threshold (minimum number of annotators agreeing) increased. As previously mentioned, a consensus variation only occurred if the consensus threshold exceeded the actual number of agreeing annotators for a given pixel and at least one of them disagreed. This ensured that we only considered actual pixel-wise classification differences and not the subjective choice to not annotate an image region.

The plot suggests that the consensus levels on some layers were low; for example, the minimum average agreement was only about 28% of the annotated pixels for the scleral spur.

It is also worth noticing that, although the consensus for the cornea and iris root converged to an almost stable percentage for high consensus threshold values, the consensus for the trabecular meshwork, scleral spur, and ciliary body band kept decreasing approximately linearly (for thresholds ≥ 2).

### Average Per-Layer Agreement Analysis

We compared the ground truth provided by every pair of participants, generating a set of 5 × 5 confusion matrices, given that five was the number of ICA layers considered.

The cell *C_i,j_* of a confusion matrix gives the number of pixels that belong to the intersection between the annotation of target *i* by the first annotator and the annotation of target *j* by the second annotator, taken as reference. Perfect agreement would result in a diagonal confusion matrix.

We computed three layer-wise metrics of inter-annotator agreement from each confusion matrix: precision, sensitivity, and Dice score. We then computed per-annotator mean values and standard deviations.

**Figure 6.**
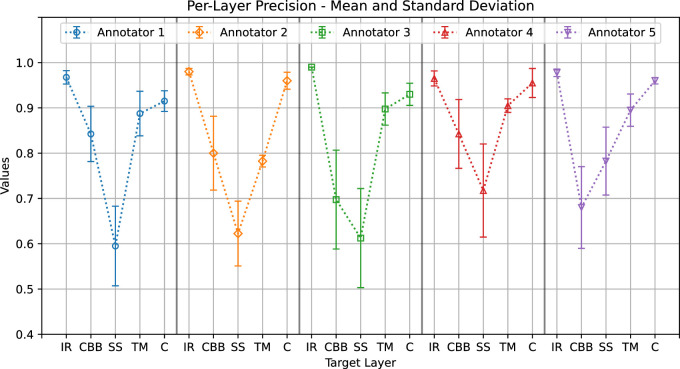
Annotators’ average precision (*plot points*) and standard deviation (*whiskers*) when annotating each layer.

The annotators’ mean precision and standard deviation values for each layer are reported in Figure 6. The ciliary body band and scleral spur overall show the lowest mean precision values and/or the highest standard deviations.

**Figure 7.**
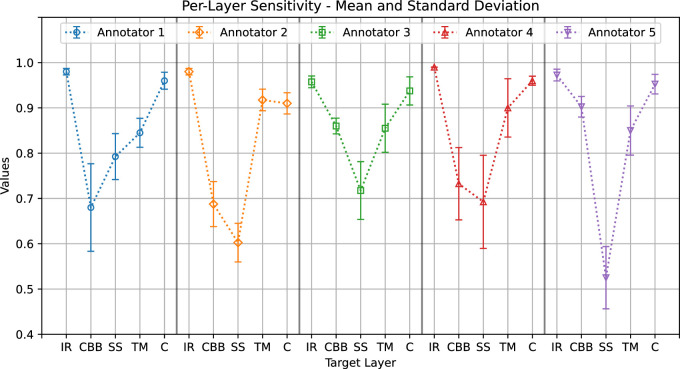
Annotators’ average sensitivity (*plot points*) and standard deviation (*whiskers*) when annotating each layer.

Figure 7 shows the mean sensitivity values and standard deviations. As in the case of precision, the maximum overall variability occurred for the ciliary body band and scleral spur. Comparing Figures 6 and 7 gives us an insight into the differences between annotations from different clinicians. For a specific annotator and target, good precision but low sensitivity suggests that, compared with contours traced by others, the area delineated was thinner but centered on average, thus generating a prevalence of false-negative classifications (e.g., for annotator 1, ciliary body band). Good sensitivity but lower precision suggests that the delineated area was larger but centered on average, generating a prevalence of false-positive classifications (e.g., for annotator 5, ciliary body band). Low precision and sensitivity suggest that the delineated area was displaced from the average (e.g., for annotator 2, scleral spur).

**Figure 8.**
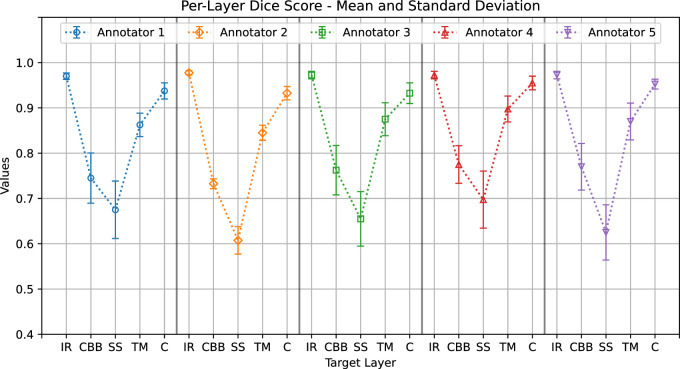
Annotators’ average Dice score (*plot points*) and standard deviation (*whiskers*) when annotating each layer.

Figure 8 shows the mean Dice scores and corresponding standard deviation values, providing a quantitative metric to compare annotations from different clinicians.

The graphs show a common pattern: good agreement on the iris root, trabecular meshwork, and cornea (with average Dice score ranges of 0.97–0.98, 0.84–0.9, and 0.93–0.96, respectively) and lower agreement on the ciliary body band and scleral spur (with average Dice score ranges of 0.61–0.7 and 0.73–0.78, respectively).

**Figure 9.**
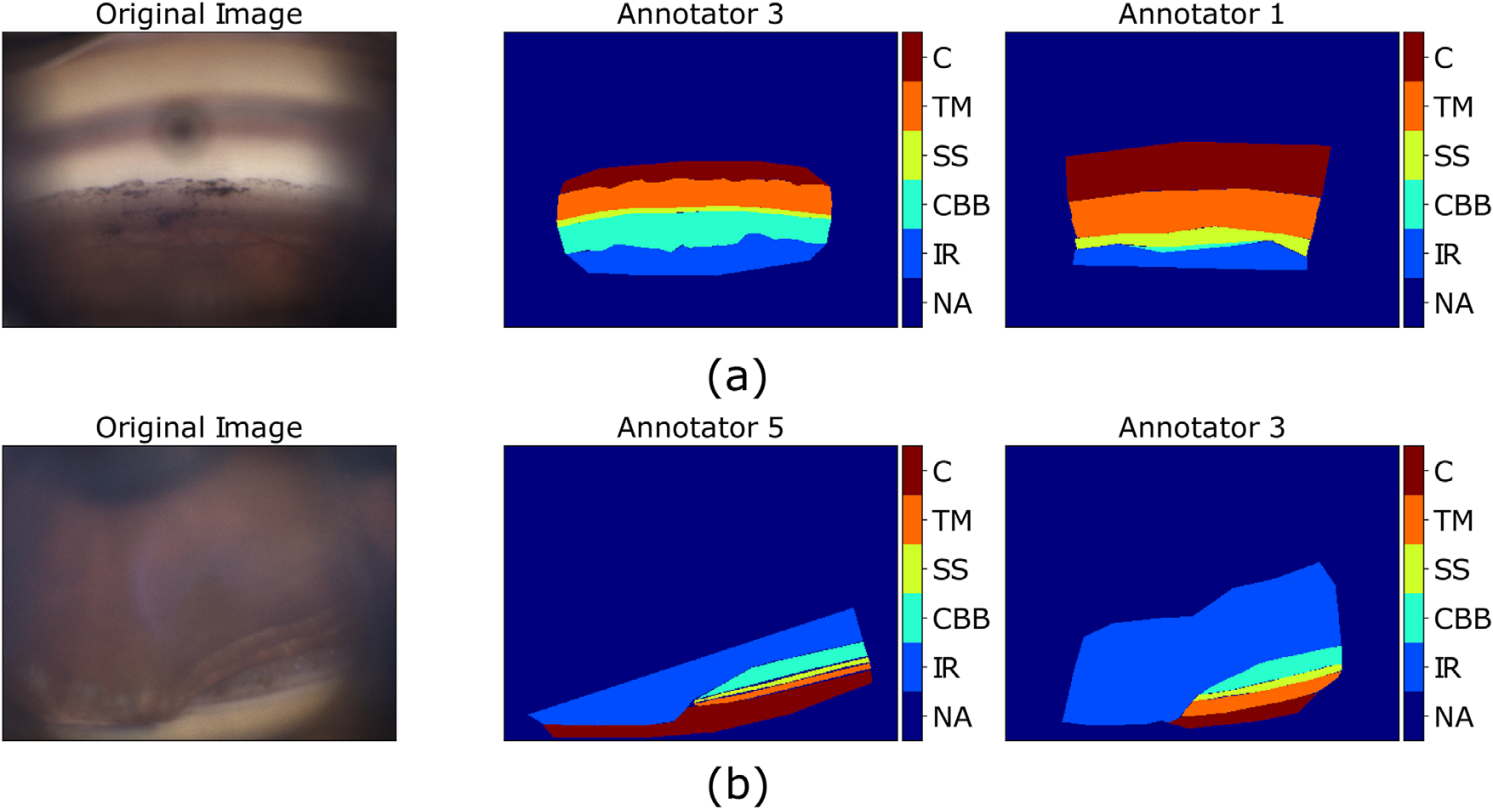
Visual representation of cases that led to low agreement metric values. (a) Low-precision CBB annotation and low Dice score SS annotation. (b) Low-sensitivity TM annotation. (Gonio photographs courtesy of C.E. Traverso, MD, Clinica Oculistica Di.N.O.G.M.I., Ospedale San Martino, Genoa, Italy).

Figure 9 shows two examples of annotations that led to low values of per-layer agreement metrics. Figure 9a compares annotator 3 and annotator 1. The ciliary body band annotation provided by annotator 3 included that of annotator 1, but it was larger and generated false positives in regions annotated differently by annotator 1 and, in turn, a low precision score. In the same comparison, the two scleral spur annotations cover different regions of the image, thus returning a low Dice score (low sensitivity and low precision). Figure 9b compares annotator 5 with annotator 3. The trabecular meshwork annotation of annotator 5 included that of annotator 3 but it was thinner, which caused false negatives in part of the region annotated as cornea by annotator 5, thus returning a low sensitivity score.

## Discussion

Well-designed data annotations are a crucial component of the development of reliable machine learning algorithms. When annotations from different experts are available, modeling their variability is important to interpret algorithm performance correctly, especially in the field of medical data analysis, where it is often impossible to obtain objective ground truth.

In this work, to the best of our knowledge, we have presented the first inter-observer variability study on segmentations of clinically relevant anatomical layers in digital images of the ICA.

The data annotation protocol and the study itself have been designed to support the development of deep learning algorithms to perform automated gonio photographs processing, with a particular focus on deep learning for semantic segmentation of layers interfaces. Automatic systems for layer segmentation could assist with the assessment of local image features, such as synechial closure extension and its changes over time, which would not be possible without pixel-wise annotations of image data.

From the analysis of the annotations provided by five experienced ophthalmologists we obtained a detailed, quantitative description of the inter-annotator variability that can be summarized in the following points:

1. Providing contours of ICA structures in digital gonio photographs is challenging due to target feature variability (e.g., pigmentation, color shades) and image quality (e.g., illumination, sharpness, focus). This led to differences in the number of times the participants felt sufficiently confident to delineate target structures even where their presence was expected from anatomical knowledge.
2. The consensus area of per-layer segmentation regions, defined as the number of pixels labeled the same by a minimum number of annotators, was much smaller for the scleral spur and ciliary body band compared with other layers (only about 28% and 41% of the pixels annotated as such by at least one expert). This result is particularly relevant because the scleral spur is an important marker to classify an ICA as fully open.
3. The average values of agreement metrics showed a common pattern among annotators. High agreement values were found for structures with boundaries better characterized in terms of visual features of the images (e.g., contrast, color, texture)—namely, the iris root, trabecular meshwork, and cornea. Low agreement values were found for the ciliary body band and scleral spur regions.

Our findings suggest that inter-annotator variability is generally lower in appositional angle-closure images than in open-angle images. The configuration of visible layers (i.e., iris root and cornea) in appositional angle-closure images is simpler, and only the direct iridocorneal interface is a possible source of variability. In our digital gonio photographs, the iridocorneal interface in appositional angle-closure images is a sharp boundary and was generally delineated consistently by annotators. For this reason, we do not expect that the inclusion of more appositional angle closure images in the dataset would increase the overall inter-annotator variability.

The current study has some limitations, in particular the limited numbers of images and ophthalmologists involved, although many papers in the literature of ophthalmic image analysis report experiments involving up to only three or four annotators. The reason for this is that generating annotations is time consuming, and clinical time is at a premium.

Nevertheless, our results provide important information on inter-annotator variability at delineating anatomical layers of the ICA in digital gonio photographs, at least in two ways. First, they provide a quantitative context for interpreting values of assessment measures obtained when validating automatic systems. Second, they give a first insight into the consensus of clinicians analyzing digital gonio photographs, which seems clearly dependent on specific layers. Given the variability of annotations by different experts, training and validating systems for automated gonioscopy with data acquired from several annotators seems strongly advisable to improve generalization. Estimating output uncertainty is necessary to highlight image features that are more difficult to classify (and possibly linked to increased inter-annotator variability), thus improving interpretability and ultimately clinicians’ trust in these algorithms.

Larger studies are advisable to firm up our conclusions to obtain truly reliable validation of artificial intelligence and machine learning applications for computer-aided analysis of gonioscopic images in the framework of evaluation of risk factors associated with glaucoma development, categorization of the disease, and support for the choice of treatments.

In particular, automated algorithms for gonio photograph segmentation may help improve gonioscopy repeatability and provide an efficient baseline processing method for the automatic extraction of clinical parameters.
